# Glucocorticoid excess alters metabolic rate and substrate utilisation via 11β-HSD1

**DOI:** 10.1530/JOE-24-0205

**Published:** 2024-10-28

**Authors:** Samuel R Heaselgrave, Silke Heising, Stuart A Morgan, David M Carthwright, Michael Sagmeister, Rowan S Hardy, Craig L Doig, Nicholas Morton, Kostas Tsintzas, Gareth G Lavery

**Affiliations:** 1 Center of Hypothalamic Research, Department of Internal Medicine, University of Texas Southwestern Medical Center, Dallas, Texas, USA; 2 Centre for Systems Health and Integrated Metabolic Research, Department of Biosciences, Nottingham Trent University, Nottingham, UK; 3 Institute of Metabolism and Systems Research, University of Birmingham, Birmingham, UK; 4 Centre for Cardiovascular Science, University of Edinburgh, Edinburgh, UK; 5 MRC Versus Arthritis Centre for Musculoskeletal Ageing Research, School of Life Sciences, University of Nottingham, Queen’s Medical Centre, Nottingham, UK

**Keywords:** 11β-HSD1, glucocorticoid excess, metabolic rate, substrate utilisation

## Abstract

Systemic glucocorticoid excess causes several adverse metabolic conditions, most notably Cushing’s syndrome. These effects are amplified by the intracellular enzyme 11β-hydroxysteroid dehydrogenase type 1 (11β-HSD1). Here, we determined the less well-characterised effects of glucocorticoid excess, and the contribution of 11β-HSD1 amplification on metabolic rate in mice. Male and female C57BL/6J (wild type, WT) and 11β-HSD1 knockout (11β-HSD1 KO) mice were treated with high-dose corticosterone or a vehicle control for 3 weeks. Indirect calorimetry was conducted during the final week of treatment, with or without fasting, to determine the impact on metabolic rate. We found that corticosterone treatment elevated metabolic rate and promoted carbohydrate utilisation primarily in female WT mice, with effects more pronounced during the light phase. Corticosterone treatment also resulted in greater fat accumulation in female WT mice. Corticosterone induced hyperphagia was identified as a likely causal factor altering the respiratory exchange ratio (RER) but not energy expenditure (EE). Male and female 11β-HSD1 KO mice were protected against these effects. We identify novel metabolic consequences of sustained glucocorticoid excess, identify a key mechanism of hyperphagia, and demonstrate that 11β-HSD1 is required to manifest the full metabolic derangement.

## Introduction

Glucocorticoids (GCs) are a class of steroid hormones critical for whole-body energy homeostasis (Tomlinson & Stewart 2001). GCs serve as stress response hormones driving GC receptor-mediated transcription to alter metabolic processes in response to endogenous or exogenous stimuli (Nicolaides *et al.* 2015). Given their metabolic influence across a range of tissues (Magomedova & Cummins 2016) systemic glucocorticoid levels are tightly governed by the hypothalamus–pituitary–adrenal axis (Ramamoorthy & Cidlowski 2016). Despite this regulatory mechanism, endogenous glucocorticoid excess can occur. However, more commonly, iatrogenic-induced hypercortisolaemia occurs (Barbot *et al.* 2020). Sustained excess results in metabolic dysfunction, most notably Cushing’s syndrome, which is characterised by a well-documented phenotype in humans and rodents (Lacroix *et al.* 2015, Lonser *et al.* 2017). This phenotype, which can be readily induced in mice for experimental purposes (Karatsoreos *et al.* 2010, Morgan *et al.* 2014, Uehara *et al.* 2020, Nishiyama *et al.* 2022), incorporates numerous metabolic features such as loss of glucose sensitivity, hypertension, hepatic steatosis, myopathy, obesity, and skeletal muscle atrophy (Schakman *et al.* 2008, Goodwin & Geller 2012, Di Dalmazi *et al.* 2012, Abraham *et al.* 2013, Woods *et al.* 2015, Morgan *et al.* 2016). Many of these metabolic features of Cushing’s syndrome are underpinned at the pre-receptor level by the enzyme 11β-hydroxysteroid dehydrogenase type 1 (11β-HSD1) (Tomlinson *et al.* 2002, Morgan *et al.* 2014). In humans, this involves the conversion of inactive cortisone to active cortisol (11 dehydro-corticosterone and corticosterone in rodents) (Tomlinson & Stewart 2001). Mice with 11β-HSD1 gene deletion (11β-HSD1 KO) and humans born lacking functional 11β-HSD1 are protected from the metabolic consequences of glucocorticoid excess (Tomlinson *et al.* 2002, Morgan *et al.* 2014).

Whilst there are many studies documenting the metabolic complications of glucocorticoid excess, there remain deficits in our understanding of impacts upon whole-body metabolic rate and substrate utilisation. These are defined, respectively, by energy expenditure (EE: indirectly calculated using oxygen consumption and carbon dioxide production values) and the respiratory exchange ratio (RER: calculated by dividing carbon dioxide production by oxygen consumption, giving values that allow an estimation of carbohydrate versus fatty acid oxidation in the organism). Normal fluctuations in endogenous glucocorticoid production were reported to have no effect on metabolic rate in mice (Dlugosz *et al.* 2012) or humans (Jobin *et al.* 1996). However, elevated EE, oxygen consumption, carbon dioxide production, and altered RER were reported following acute or short-term low-dose exogenous glucocorticoid treatment in humans (Bessey *et al.* 1984, Chong *et al.* 1994, Brillon *et al.* 1995, Tataranni *et al.* 1996). Often, the effects of acute low-dose glucocorticoid treatment are not formally reported or are incomplete (Bessey *et al.* 1984, Brillon *et al.* 1995). Others have reported a decrease in metabolic rate or dispute the direction of altered substrate utilisation (Horber *et al.* 1991, Gravholt *et al.* 2002, Short *et al.* 2004, Radhakutty *et al.* 2016). Fewer studies report the effects of chronic glucocorticoid administration. Thus, chronic low-dose exogenous glucocorticoid treatment had no effect on EE in humans (Radhakutty *et al.* 2016), but decreased EE and RER in mice (Poggioli *et al.* 2013). Patients with Cushing’s syndrome were reported to have normal basal EE for their body mass and composition (Burt *et al.* 2006). Further research is required to clarify these discrepancies.

Here, we establish the effect of sustained glucocorticoid excess on whole-body metabolic rate and substrate utilisation in male and female mice, and explore what might cause any effects, using an established model of exogenous glucocorticoid excess that is known to replicate the metabolic impacts seen in humans and those with Cushing’s syndrome (Morgan *et al.* 2014, Fenton *et al.* 2019). We also determine if any effects are mediated by the enzyme 11β-HSD1.

## Materials and methods

### Animals and glucocorticoid administration

Male and female C57BL/6J (wild type, WT) mice were purchased from Charles River, UK. All 11β-HSD1 KO mice were homozygous and bred in-house on a C57BL/6J background. Mice were group-housed in sex- and litter-matched cages in groups of 2–4. Cages were kept at a standard temperature (22°C) in a humidity-controlled environment with a uniform 12-h light:darkness cycle. Mice were individually housed during the final 120 h of indirect calorimetry assessment. Throughout, mice were provided with nesting material, with food and water available *ad libitum*. Mice were given a standard chow diet (EURodent Diet 14%, Labdiet, St. Louis, MO, USA), though the water differed depending on the treatment group. Treatment lasted for 3 weeks and utilised an established protocol known to induce a phenotype typical of sustained glucocorticoid excess (Morgan *et al.* 2014, Fenton *et al.* 2019). Drinking water containing 100 mg/L corticosterone (Sigma-Aldrich) or a vehicle control (0.6% ethanol) was given *ad libitum*. All mice were 10 weeks of age at the start of treatment. Mice were culled immediately upon completion of indirect calorimetry assessment. All animal procedures were conducted in accordance with UK Home Office regulations, the UK Animals (Scientific Procedures) Act 1986, and were approved locally by the University of Birmingham and Nottingham Trent University AWERB committees under the project licence number PP1816482.

### Indirect calorimetry

Initial indirect calorimetry was performed during the final week of treatment using a TSE PhenoMaster 8 cage system (TSE Systems, Bad Homburg, Germany). Mice were group-housed for 48 h in the PhenoMaster to acclimatise. Mice were then individually housed for 120 h within the PhenoMaster. The first 24 h of which were to acclimate the mice to isolation, whilst the subsequent 96 h were for undisturbed data collection. Markers of metabolic rate and substrate utilisation (EE and RER) were measured. Food and water intake were also measured. All measurements were recorded in male and female WT (*n* = 12) and 11β-HSD1 KO mice (*n* = 5–8). Subsequently, locomotor activity was assessed in female WT mice only (*n* = 8). Further indirect calorimetry assessment was performed on female WT mice only (*n* = 8) with food withdrawn during the final 12-h light phase to assess the impact of glucocorticoid excess-induced hyperphagia on the previously described markers of metabolic rate and substrate utilisation.

### Statistical analysis

Statistical significance was tested using GraphPad Prism version 9 (GraphPad Software, LLC) as well as CalR (version 1.3) (Mina *et al.* 2018). Two-way analysis of variances, followed by Tukey’s multiple comparisons test, were used to analyse all indirect calorimetry measures, and physical characteristics, and compare WT and 11β-HSD1 KO mice. Data are presented as mean ± s.e.m. throughout.

## Results

### Corticosterone treatment induces a Cushing’s phenotype in WT but not 11β-HSD1 KO mice

**Figure 1:**
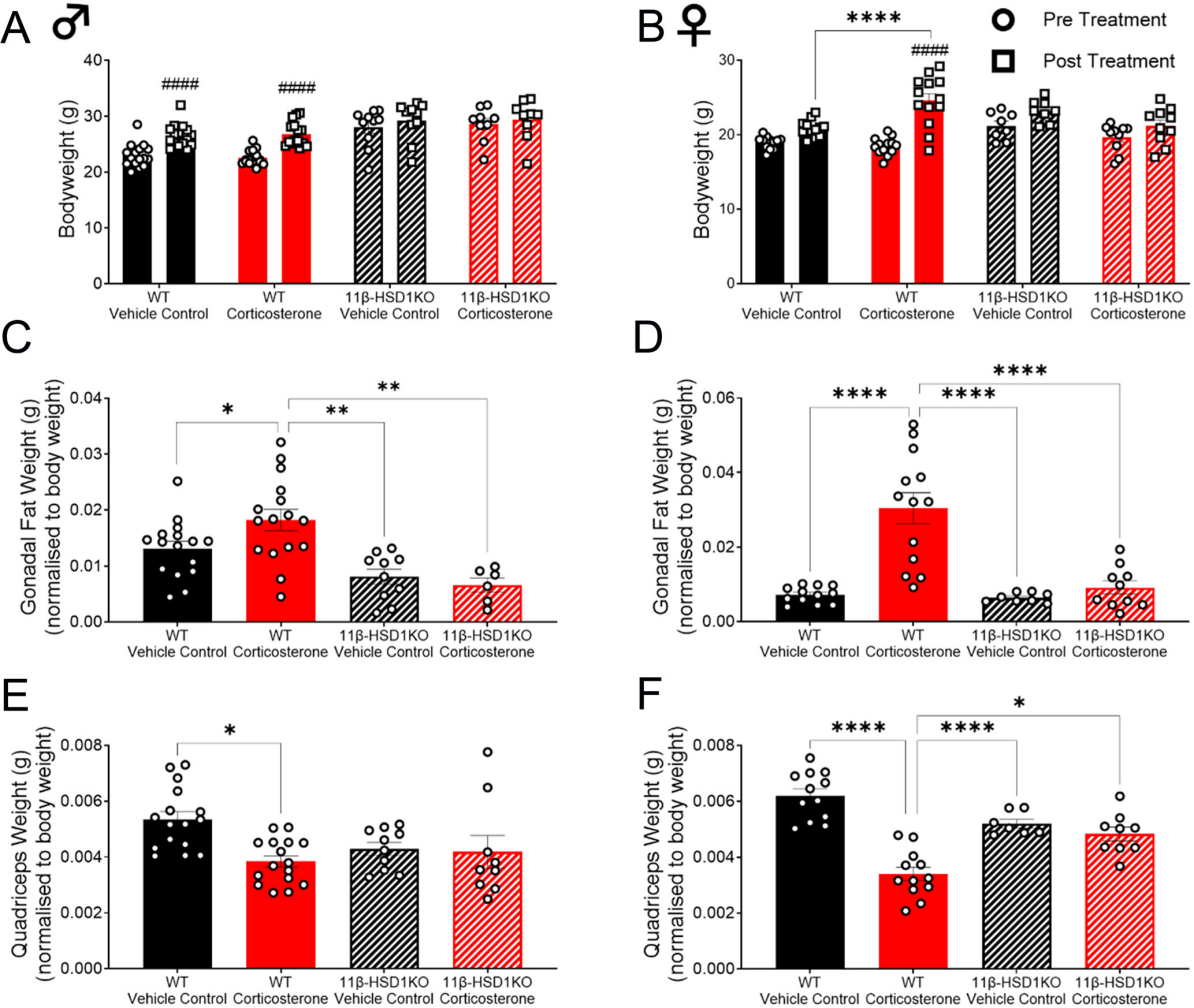
Corticosterone induced a phenotype typical of glucocorticoid excess in WT mice whilst 11β-HSD1 KO prevented it. (A) Male body weight pre- and post-treatment. (B) Female body weight pre- and post-treatment. (C) Male gonadal fat weight normalised to body weight. (D) Female gonadal fat weight normalised to body weight. (E) Male quadriceps weight normalised to body weight. (F) Female quadriceps weight normalised to body weight. Bar graphs are presented as mean ± s.e.m., *n* = 8–16. *Significant difference between treatments. # Significant pre and post treatment difference *P < 0.05, **P < 0.01, ***P < 0.001, ****/####*P* < 0.0001.

**Figure 2:**
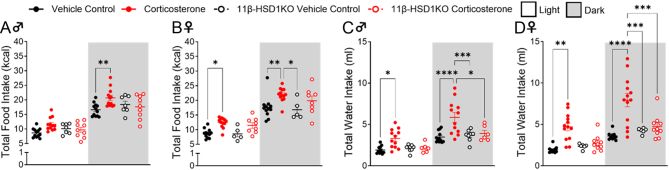
Corticosterone treatment increased food and water intake in WT mice whilst 11β-HSD1 KO prevented it. (A) Average male food intake. (B) Average female food intake. (C) Average male water intake. (D) Average female water intake. Scatter plots represent average day and night values and are resented as mean with individual values ± s.e.m., *n* = 5–12. *Significantly different. *P < 0.05, **P < 0.01, ***P < 0.001, ****P < 0.0001.

To assess the impact of glucocorticoid excess on metabolic rate and substrate utilisation, we first confirmed the development of the known Cushing’s phenotype in WT mice. As expected, corticosterone treatment caused male and female WT mice to develop these signs (Fig. 1). Increased lipid accumulation (Fig. 1C and D) (Peckett *et al.* 2011, Gasparini *et al.* 2016) and muscle atrophy/reduced lean body mass accrual (Fig. 1E and F) (Schakman *et al.* 2013, Sato *et al.* 2018, Gasparini *et al.* 2019) were demonstrated. This occurred despite a significant increase in control male WT body weight and a trend towards increased control female WT bodyweight (*P* = 0.07) over the duration of the study. Polydipsia (Tataranni *et al.* 1996) was observed throughout in corticosterone-treated WT mice, whilst hyperphagia (Tataranni *et al.* 1996) was continuous in females and evident during the dark phase in males (Fig. 2). The Cushingoid phenotype was more prominent in female WT mice, as they exhibited greater body weight and fat accumulation (Fig. 1B and D), as well as both hyperphagia and polydipsia (Fig. 2), compared to corticosterone-treated male mice. Successful knockout of 11β-HSD1, and therefore protection from intracellular glucocorticoid reactivation, was affirmed by the absence of this physical phenotype in male and female 11β-HSD1 KO mice (Morgan *et al.* 2014) (Fig. 1). Corticosterone-induced polydipsia was also prevented, whilst hyperphagia was attenuated in 11β-HSD1 KO mice (Fig. 2).

### Metabolic rate is elevated in a sex-specific manner by glucocorticoid excess in WT but not 11β-HSD1 KO mice

**Figure 3:**
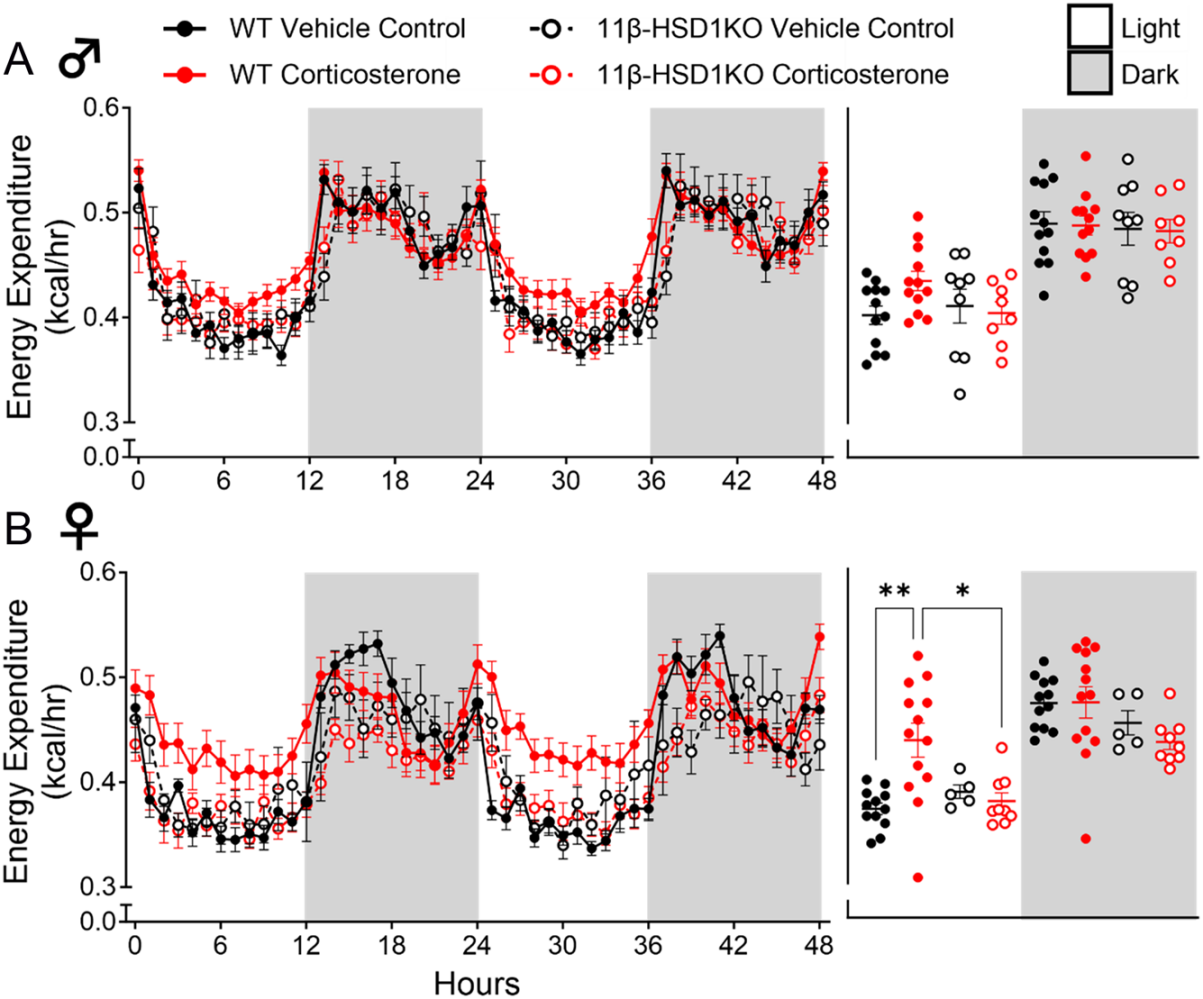
Glucocorticoid excess elevated energy expenditure in female WT mice, whilst 11β-HSD1 KO prevented this. (A) Male energy expenditure. (B) Female energy expenditure. Line graphs represent hourly change and are presented as mean ± s.e.m., *n *= 12. Scatter plots represent average day and night values and are presented as mean with individual values ± s.e.m., *n* = 12. *Significantly different. *P < 0.05, **P < 0.01.

**Figure 4:**
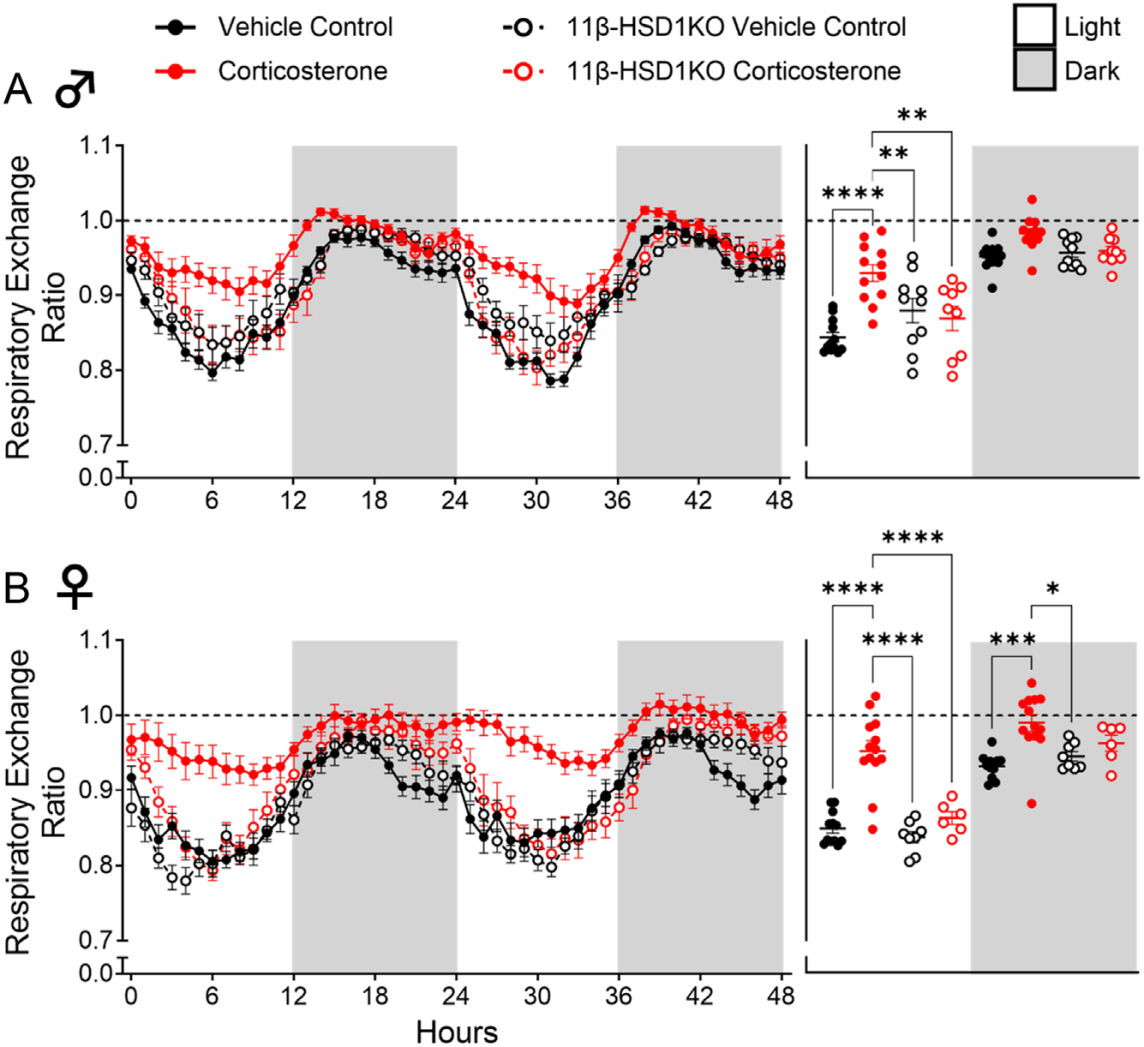
Glucocorticoid excess altered the respiratory exchange ratio in female WT mice, whilst 11β-HSD1 KO prevented this. (A) Male respiratory exchange ratio. (B) Female respiratory exchange ratio. Line graphs represent hourly change and are presented as mean ± s.e.m., *n* = 12. Scatter plots represent average day and night values and are presented as mean with individual values ± s.e.m., *n* = 12. *Significantly different. *P < 0.05, **P < 0.01, ***P < 0.001, ****P < 0.0001.

The degree to which glucocorticoid excess impacts mammalian energy metabolism remains unclear (Jimeno & Verhulst 2023). We sought to provide whole-body indirect calorimetric analysis of glucocorticoid excess-exposed WT mice. Corticosterone treatment did not significantly alter EE (Fig. 3A) in male WT mice. However, it did significantly increase the RER during the light phase (Fig. 4A). In female WT mice, corticosterone significantly elevated EE during the light phase (Fig. 3B), and increased RER during the light and darkness phases (Fig. 4B). This suggests increased calorie usage in female mice, which was driven predominantly by increased carbohydrate utilisation, an effect experienced by both females and males. Notably, corticosterone caused average RER to approach or exceed a value of 1.0 during the dark phase in males and females (Fig. 4), effects indicative of an increased rate of *de novo* lipogenesis (Solinas *et al.* 2015). Assessment in 11β-HSD1 KO mice revealed further protection against glucocorticoid excess as corticosterone treatment did not elevate EE or change substrate utilisation (RER) in male or female 11β-HSD1 KO mice (Figs. 3 and 4).

### Physical activity was decreased by glucocorticoid excess in female WT mice

**Figure 5:**
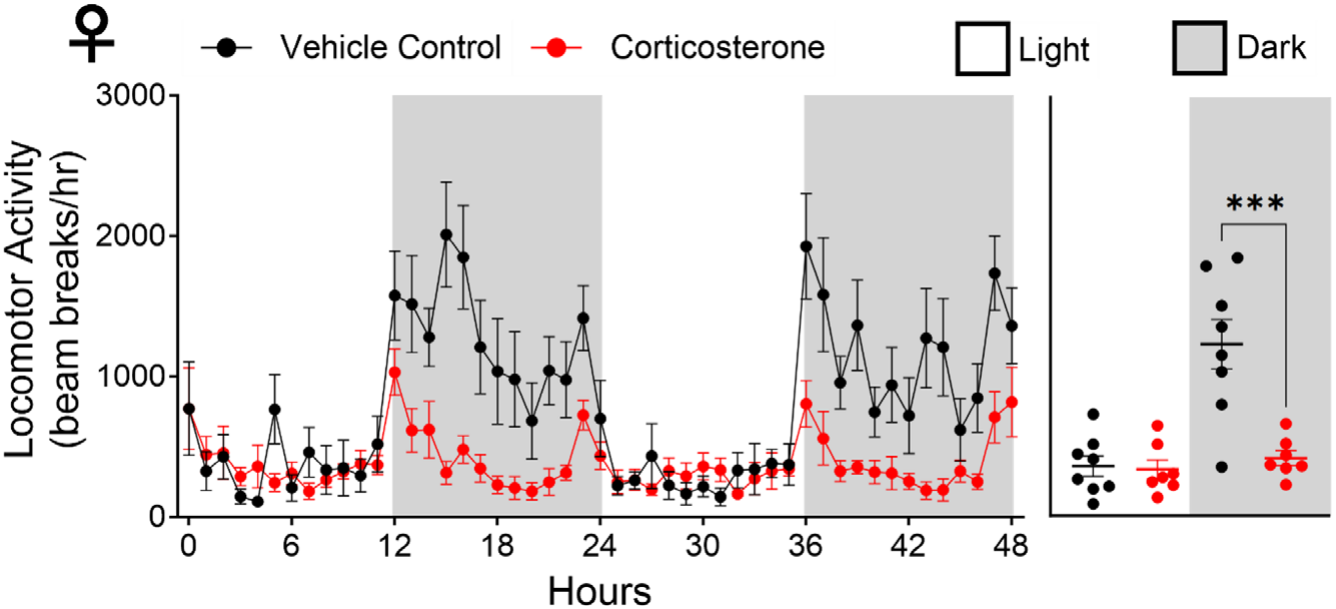
Glucocorticoid excess decreased physical activity (locomotor activity) levels in female WT mice. The line graph represents hourly changes and are presented as mean ± s.e.m., *n *= 8. The scatter plot represents average day and night values and are presented as mean with individual values ± s.e.m., *n* = 8. *significantly different. ***P < 0.001.

Sexual dimorphism is a widely appreciated characteristic of glucocorticoid function, as both androgens and oestrogens can modulate GC transcriptional activity, which can also directly influence endogenous glucocorticoid synthesis (Takahashi *et al.* 2024). Given our observed increase in female EE, we set out to explore the effects of corticosterone on physical activity, as increased physical activity is known to increase EE (van Baak 1999). This was explored in female WT mice only due to their exaggerated corticosterone responses. Locomotor activity, a definitive measure of physical activity, was assessed. Locomotor activity (Fig. 5) was decreased by corticosterone treatment during the dark phase, resulting in sustained sedentary behaviour across both the light and darkness phases which ultimately revealed a corticosterone-induced disconnect between physical activity and EE.

### Food withdrawal normalised the respiratory exchange ratio but not energy expenditure in female WT mice

**Figure 6:**
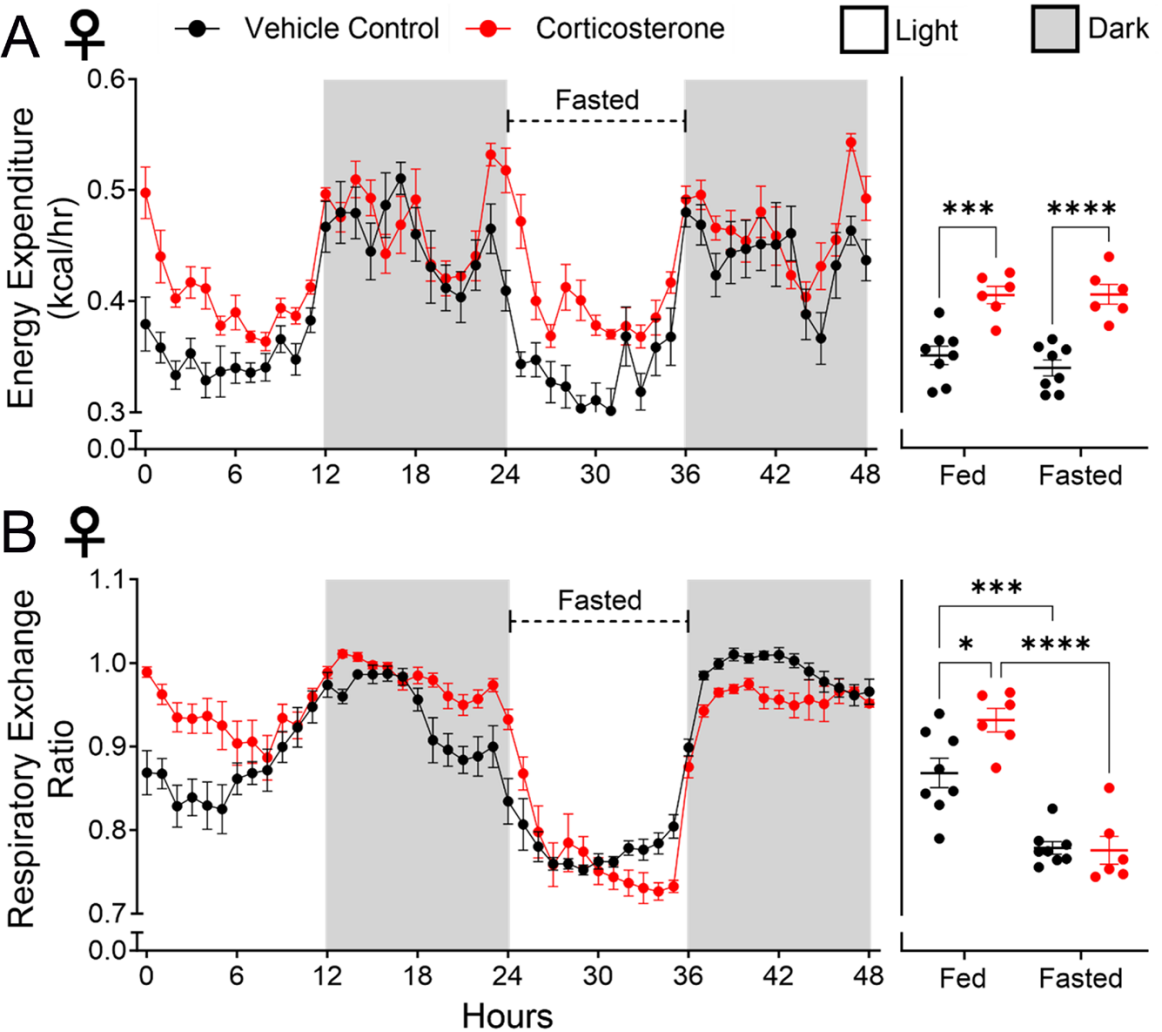
Fasting during the light phase prevented an altered respiratory exchange ratio but did not prevent elevated energy expenditure in female WT mice. (A) Energy expenditure. (B) Respiratory exchange ratio. Line graphs represent hourly change and are presented as mean ± s.e.m., *n* = 8. Scatter plots represent average day and night values and are presented as mean with individual values ± s.e.m., *n* = 8. *Significantly different. *P < 0.05, **P < 0.01, ***P < 0.001, ****P < 0.0001.

Sustained excess corticosterone exposure is associated with hyperphagia (Sefton *et al.* 2016). Therefore, increased food intake and the subsequent thermic effect of food were considered possible explanations for both elevated EE and altered RER measured in the female mice (Solinas *et al.* 2015, Ho 2018). To evaluate this, female WT mice were fasted for a 12-h light phase. Fasting had no effect on EE, which remained elevated and did not differ from the previous fed 12-h light phase (Fig. 6A). This revealed that corticosterone-induced elevations in EE were not an artefact of the hyperphagia it promotes. Fasting, however, reversed the elevation of RER values seen in corticosterone-treated mice (Fig. 6B), suggesting that the corticosterone-treated mice maintained an ability to utilise lipids for fuel when necessary.

## Discussion

Knowledge of the deleterious effects of sustained glucocorticoid excess is well understood but remains incomplete at the level of whole-body physiology. Herein, we demonstrate that glucocorticoid excess elevates metabolic rate (EE) and alters substrate utilisation (RER) in WT mice with greater significance in females than males. Notably, corticosterone suppresses physical activity in female WT mice, whilst light-phase fasting fails to prevent elevated EE. This suggests that neither increased energy of movement nor digestion is responsible. Hyperphagia and lipogenesis likely promote altered RER and are compatible with increased adipose accumulation. RERs exceeding 1.0 are also a signature of lipogenesis (Solinas *et al.* 2015). Critically, these effects are dependent upon the presence of 11β-HSD1, revealing further protection against glucocorticoid excess from disruption of 11β-HSD1 function.

A strong causal candidate for increased EE is canonical activation of brown adipose tissue non-shivering thermogenesis (Cannon & Nedergaard 2004), which occurs at a tonic level at room temperature in mice (Bastias-Perez *et al.* 2020). This is unlikely to be responsible, as there is strong evidence that rodents exposed to glucocorticoid excess exhibit profound suppression of the thermogenic protein UCP1 (Poggioli *et al.* 2013, Doig *et al.* 2017, Kaikaew *et al.* 2019). An alternative thermogenic mechanism, futile creatine cycling, is also unlikely to explain our findings, as this process is suppressed in inactive, hyperphagic mice (Hepler *et al.* 2022). Increased physical activity is another potential explanation (Van Baak 1999) that can be eliminated, as corticosterone-treated WT mice were continuously inactive. Similar to activity, the thermic effect of food requires examination (Tataranni *et al.* 1996, Solinas *et al.* 2015, Ho 2018) but is unlikely to be responsible for these measurements, as fasting failed to prevent increased EE. Therefore, increased body weight, driven by increased adiposity, is a likely explanation for GC-induced shifts in female EE. This agrees with the biological interpretation of Newton’s second law, which determines that an animal of greater mass is required to burn more calories to meet increased energy demands (Corrigan *et al.* 2020). The more significant EE elevation seen in female mice might also be explained by this demand, as their body weight increase was greater than that of male mice.

Elevated RER, close to or exceeding 1.0, indicates that sustained corticosterone exposure forces WT mice to primarily utilise carbohydrates as their fuel source, at the expense of lipid utilisation. It also indicates some mice undergo increased *de novo* lipogenesis, which can be attributed to glucocorticoid excess and hyperphagia of their 76% carbohydrate-based diet (Magomedova & Cummins 2016, Talal *et al.* 2021). Increased *de novo* lipogenesis in female mice, as indicated by a greater RER increase and reported in female mice given a high carbohydrate diet (Low *et al.* 2018), likely contributes to their greater adipose accrual. Given this, it was hypothesised that hyperphagia of a carbohydrate-based diet was elevating RER. Fasting of mice supported this, as RER decreased towards 0.7, indicating increased lipid utilisation. Therefore, glucocorticoid excess does not directly restrict mice to carbohydrate utilisation, but the glucocorticoid-induced hyperphagia does. Whether different diets, such as those low in carbohydrates, might attenuate the impact of this hyperphagia merits investigation, as a low-carbohydrate diet can aid in the management of Cushing’s syndrome (Dugandzic *et al.* 2022).

Sex differences in mice treated with corticosterone, in the context of indirect calorimetry, are novel. A greater increase in body weight driven by a greater accumulation of adipose tissue potentially explains the greater EE elevation in female mice. Whilst WT male control mice significantly grew over the course of treatment, and females did not, potentially limiting the relative increase in male body weight and adiposity from corticosterone treatment, it is unlikely this confounded the observed sex differences as WT female control mice in fact trended towards increased body weight over the course of treatment (*P* = 0.07). Therefore, the cause for this greater body weight and increased adiposity in female mice is more likely due to increased hyperplastic as opposed to hypertrophic expansion of adipose tissue in female mice (Kaikaew *et al.* 2019). Likely elevated *de novo* lipogenesis in female mice, as indicated by RER, is another explanation for increased adiposity. Differences might also be caused by increased 5α-reductase expression in male mice, which has the potential to increase corticosterone deactivation and clearance, potentially mitigating the impact of their orally consumed dose (Melcangi *et al.* 1998, Nixon *et al.* 2012), but this remains to be established.

The majority of literature related to human metabolic responses to GC excess focusses on acute or low-dose treatment. Of these acute studies, agreement is found with some (Bessey *et al.* 1984, Chong *et al.* 1994, Brillon *et al.* 1995, Tataranni *et al.* 1996), but not all (Horber *et al.* 1991, Gravholt *et al.* 2002, Short *et al.* 2004, Radhakutty *et al.* 2016). However, due to the acute nature of these studies, meaningful comparisons are limited. Of the existing chronic studies, no agreement is found. The only existing mouse study, to the authors' knowledge, reported that dexamethasone treatment for 7 weeks decreased both EE and RER (Poggioli *et al.* 2013). Whereas, in chronic human investigation with rheumatoid arthritis patients, 6 months of 6 mg prednisolone a day had no effect on EE (Radhakutty *et al.* 2016). Finally, in patients with Cushing’s syndrome, EE remained unaltered (Burt *et al.* 2006). Reasons for such variability are yet to be determined. However, the use of different synthetic glucocorticoids in the literature might be contributory (Burt *et al.* 2006, Poggioli *et al.* 2013, Radhakutty *et al.* 2016). Differences in indirect calorimetry methodology between the present study and existing mice (Poggioli *et al.* 2013) and human studies (Burt *et al.* 2006, Radhakutty *et al.* 2016) might also be responsible. Additionally, variation might indicate species-specific effects. Finally, as the present study rapidly induced a Cushingoid phenotype, but the other three studies utilised more prolonged glucocorticoid exposure, the length of treatment must also be considered.

This study revealed that 11β-HSD1 KO prevents elevation of EE and altered RER in mice exposed to glucocorticoid excess. Additionally, it attenuates hyperphagia and polydipsia in male and female mice. Interestingly, however, control-treated 11β-HSD1 KO mice did not display comparable growth rates to WT control mice, contrary to existing literature (Morgan *et al.* 2022). Whilst this is unlikely to have confounded the findings, as 11β-HSD1 KO responded as expected to corticosterone treatment with attenuated lipid accumulation and skeletal muscle atrophy (Morgan *et al.* 2014), it must be considered. Despite this, and despite being the first to investigate the impact on metabolic rate and substrate utilisation, the findings of the present study complement existing literature reporting 11β-HSD1 mediation of other consequences of glucocorticoid excess in mice and humans (Tomlinson *et al.* 2002, Morgan *et al.* 2014, Webster *et al.* 2021, Othonos *et al.* 2023). Therefore, altered metabolic rate and substrate utilisation might be consequences of, or causal factors in, other metabolic conditions that are caused by glucocorticoid excess and mediated by 11β-HSD1. As such, they should be assessed as part of the ongoing development of human 11β-HSD1 inhibitors (Othonos *et al.* 2023, Pofi *et al.* 2023).

**Figure 7:**
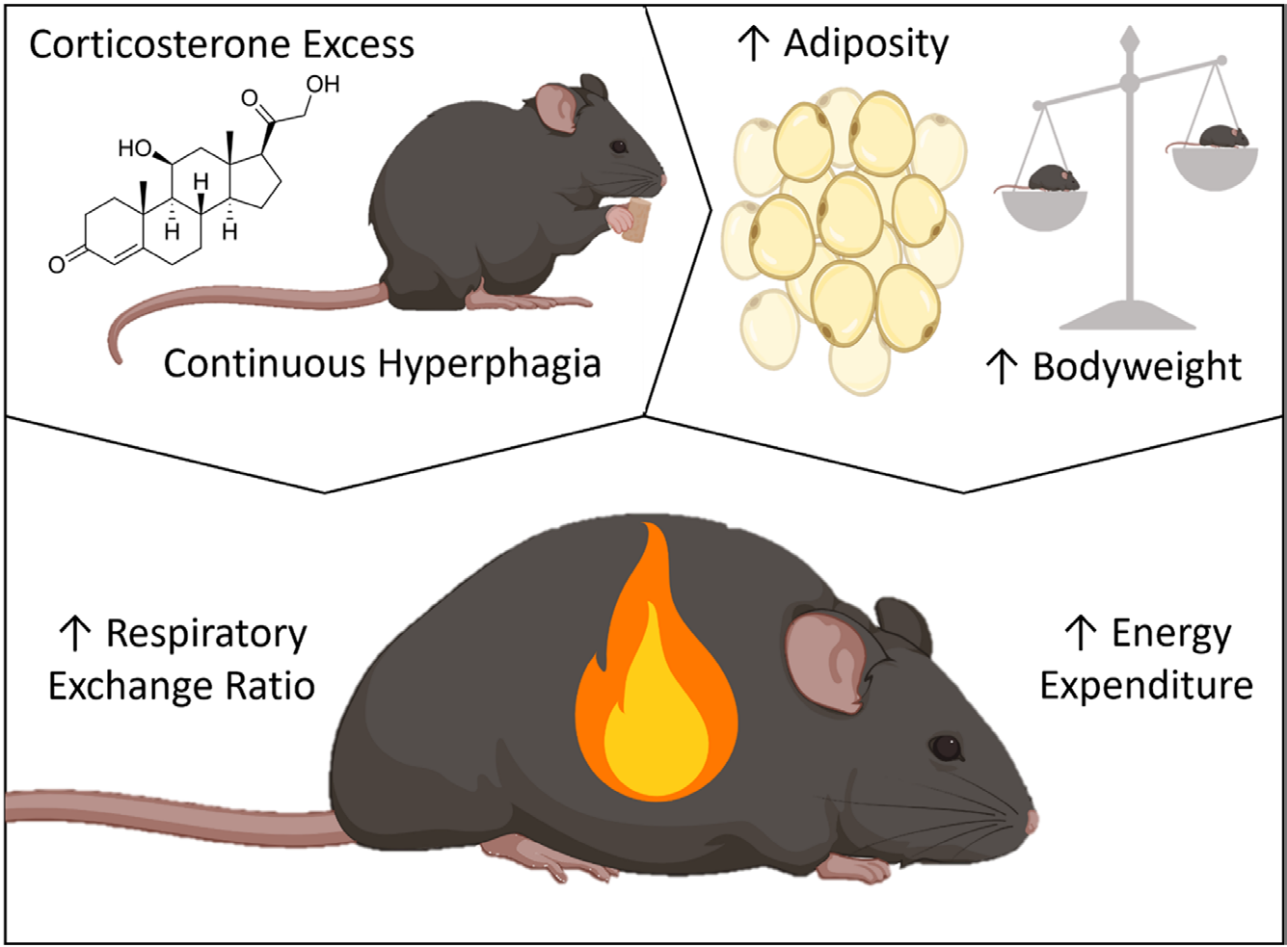
Proposed mechanism by which glucocorticoid excess elevates energy expenditure and the respiratory exchange ratio in C57BL/6J mice. Excess corticosterone treatment forces mice into a continuous state of hyperphagia, which, alongside corticosterone excess itself, causes a pronounced increase in adipose tissue. This increases body weight, which likely elevates energy expenditure. Alteration of the respiratory exchange ratio is driven directly by glucocorticoid excess-induced hyperphagia.

In conclusion, this study identifies novel metabolic consequences of glucocorticoid excess, which include elevated EE and altered RER. Findings also identify greater effects in female mice, with alterations in male mice failing to reach the same scale or significance. Hyperphagia was identified as a causal factor altering RER, whilst increased body weight driven by increased adiposity is theorised to be responsible for elevating EE (Fig. 7). These effects are mediated by 11β-HSD1, with male and female mice receiving protection from 11β-HSD1 KO. Together, these findings provide further insights into the underlying mechanisms and consequences of exogenous glucocorticoid excess.

## Declaration of interest

The authors declare that there is no conflict of interest that could be perceived as prejudicing the impartiality of the study reported.

## Funding

SRH was a recipient of a PhD scholarship funded by the MRC Versus Arthritishttp://dx.doi.org/10.13039/501100012041 Centre for Musculoskeletal Ageing Research, University of Birmingham and University of Nottingham. GGL was supported by a Wellcome Trusthttp://dx.doi.org/10.13039/100010269 Senior Fellowship (104612/Z/14/Z). CLD is supported by Defence Medical Services Research Group funding (23/24.022). MS received funding from the Medical Research Councilhttp://dx.doi.org/10.13039/501100000265 as part of a Clinical Research Training Fellowship (MR/T008172/1). RSH was supported by Versus Arthritishttp://dx.doi.org/10.13039/501100012041. Ref: 20843.

## Author contributions

SRH, SM, DC, and GGL conceptualised the study. SRH and SH carried out the study and acquisition of data. SRH carried out data analysis. MS and RSH bred and provided 11β-HSD1 KO mice. SRH, SM, CLD, NM, KT, and GGL interpreted the results and contributed to the writing of the manuscript. GGL and KT supervised the study.
